# A Novel Method to Enhance Pipeline Trajectory Determination Using Pipeline Junctions

**DOI:** 10.3390/s16040567

**Published:** 2016-04-21

**Authors:** Hussein Sahli, Naser El-Sheimy

**Affiliations:** MMSS Research Group, Geomatics Engineering Department, University of Calgary, 2500 University Dr. NW. Calgary, AB T2N 1N4, Canada; elsheimy@ucalgary.ca

**Keywords:** pipeline junctions, Kalman Filter, inertial measurement unit, PLJ, INS, PIG

## Abstract

Pipeline inspection gauges (pigs) have been used for many years to perform various maintenance operations in oil and gas pipelines. Different pipeline parameters can be inspected during the pig journey. Although pigs use many sensors to detect the required pipeline parameters, matching these data with the corresponding pipeline location is considered a very important parameter. High-end, tactical-grade inertial measurement units (IMUs) are used in pigging applications to locate the detected problems of pipeline using other sensors, and to reconstruct the trajectories of the pig. These IMUs are accurate; however, their high cost and large sizes limit their use in small diameter pipelines (8″ or less). This paper describes a new methodology for the use of MEMS-based IMUs using an extended Kalman filter (EKF) and the pipeline junctions to increase the position parameters’ accuracy and to reduce the total RMS errors even during the unavailability of above ground markers (AGMs). The results of this new proposed method using a micro-electro-mechanical systems (MEMS)-based IMU revealed that the position RMS errors were reduced by approximately 85% compared to the standard EKF solution. Therefore, this approach will enable the mapping of small diameter pipelines, which was not possible before.

## 1. Introduction

Advances in micro-electromechanical-systems (MEMS) technology combined with the miniaturization of electronics, have made it possible to produce low cost and lightweight chip-based inertial sensors. These chips are small, lightweight, reliable and they consume very little power. They have therefore found a wide spectrum of applications in the automotive sector and other industrial applications. MEMS technology, therefore, can be used to develop navigation systems that are inexpensive, small, and consume low (microwatt) power. The attractive advantages of MEMS technology have led to remarkable research progress in the field of MEMS inertial sensors. However, on the negative side, the performance currently achieved by these low cost sensors is relatively poor due to their sensor errors. The goal of this paper is henceforth to introduce new constraints for pipeline navigation to increase the position parameters’ accuracy and reduce the total RMS.

**Figure 1:**
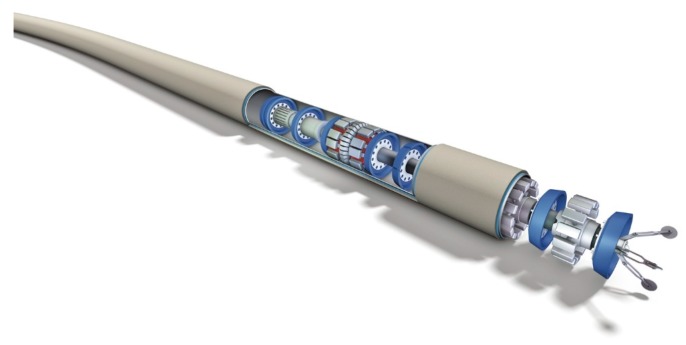
Smart Pipeline Inspection Gauge (Nord Stream AG).

Pipelines are the lifelines of a dynamic country’s infrastructure; they provide fuel, water, and all kinds of other needs that touch millions of lives. In addition, they represent one of the safest transportation methods available for crude oil, natural gas, and chemical fluids; they provide a transport medium with speed, efficiency and reliability. Furthermore, they are considered an eco-friendly option. So-called smart pipeline inspection gauges (pigs, Figure 1) commonly carry out inspection and fault identification of these pipelines.

Pigs are devices/tools that can be inserted into the pipeline and travel throughout the length of the pipeline, driven forward by the differential pressure across the tool [1,2]. Pigs carry embedded computers and sensors, to acquire information and to perform various maintenance operations in a pipeline. The pigging procedure requires the pipeline contents to be flowing to facilitate the pig’s movement. In general, the pig’s total journey length can vary from hundreds of meters to hundreds of kilometers [3]. A variety of methods are used for pipeline inspection such as ultrasonic techniques, echo sounding, radiography, and cameras. In the past, the position determination of the pig used to be achieved with a set of velocity wheels (odometers). These wheels provide the longitudinal speed of the pig that can be integrated to provide the distance traveled along the pipeline. Fiber Optic Gyro (FOG)- and/or Ring Laser Gyro (RLG)-based high-end inertial navigation systems has been proposed to be included in the positioning solution [4], which is currently being used for this purpose in the large diameter inspection tools.

This work addresses the issue of providing a MEMS-based aided inertial navigation system to replace the current high-end tactical grade IMUs. MEMS-based inertial sensors are characterized by large uncertainties and high noise (*i.e.*, bias, scale factor and non-orthogonality). Therefore, the errors in position, velocity and attitude of the motion pig grow rapidly in standalone mode. Therefore, aiding navigation systems become essential to solve the unpredictable problems of sensor errors and noises [1]. The aided information will be derived mainly from odometer sensors, pipeline modeling and Global Navigation Satellite System (GNSS).

**Figure 2:**
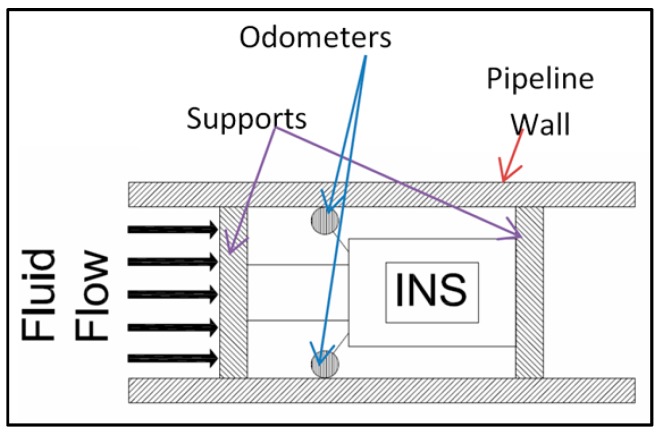
Main pig tool components.

Due to the distance underwater or underground, and/or the material that the pipeline is made of, it is not possible for the pig to communicate directly with the outside world. The collected data is saved internally and processed later (post-processed). The process requires that extra navigation sensors be used to improve the performance of the pig’s location estimation methods. A wheel odometer, for instance, can measure traveled distances (by counting the number of wheel revolutions) that are translated later into velocity measurements. These measurements can be used as external updates for the navigation algorithms. Figure 2 illustrates the main parts of the navigation sensors of a typical pig tool. Supports are mainly the front and end parts that provide the sealing for the main parts. These supports have nominal diameters larger than the pipe diameter to produce efficient sealing [2]. The pig body is contained between the two supports.

An above ground marker (AGM) is another device that can be installed on the surface of the ground above the pipeline. This device detects and records the passage of the pig in the pipeline. AGMs provide the navigation coordinates of their position (latitude, longitude, and height) along with the pig’s time passage. This information is used as a coordinate update (typically called CUPT) in the estimation algorithm. The cost of these devices often minimizes their use. Usually, these AGMs are installed every 1–3 km [5] when the pig uses high-end tactical grade IMUs.

A nonlinear sensor fusion algorithm has been applied in [6] to estimate the trajectory of pipeline pigs. IMUs along with odometers and AGMs are the sensors that have been used for navigation purposes. The filter states have been propagated using the IMU with an update from the odometer while the AGM is not reached. The AGM performs position updates when the pig passes underneath.

Similarly, in [7], an IMU along with an odometer and reference GPS have been used to simulate pig data. The results shows that by decreasing the position reference updates, the accuracy increased due to the propagation of IMU errors.

To allow proper maintenance operations and to reconstruct the pipeline trajectory in certain projects, a position referencing technique is required to find the exact coordinates of the defective parts detected by the pig’s sensors. The core of this work, therefore, is to improve the accuracy of the final reconstructed trajectories by introducing new sources of information and adding new constraints to the estimation technique.

Generally, the pig carries inertial sensors (accelerometers and gyroscopes), along with other sensors, to assess its position coordinates. To minimize possible cumulative errors arising from the MEMS-IMU, the sensor fusion technique, which involves pairing inertial sensors with non-inertial sensors, is implemented. An odometer and AGM, employed as non-inertial sensors, are used to update the algorithm.

Different estimation techniques can be used in such applications. The Extended Kalman Filter (EKF) [8,9] is commonly used for sensor fusion integration in navigation applications, especially for nonlinear dynamic models. It has been selected to demonstrate the newly proposed algorithm in this paper. In Section 2, the idea of the new algorithm will be introduced. The Methodology section (Section 3) will demonstrate the algorithm implementation, and the results will be discussed in Section 4.

## 2. New Information

### 2.1. Pipeline Junctions

**Figure 3:**
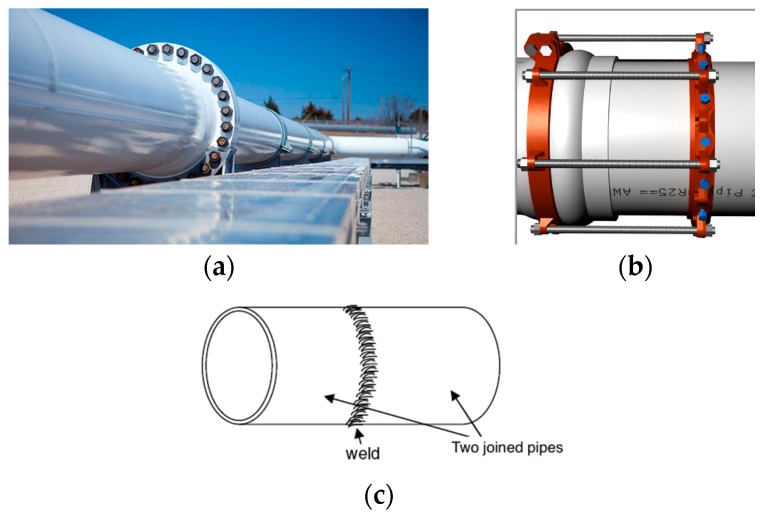
Pipeline joints: (**a**) flange type; (**b**) push-on type; (**c**) welding type.

Pipelines consist of multiple pieces of pipeline and fittings (bends, T-connections, valves … *etc.*). The pipeline pieces are fabricated in straight-line shapes. Different methods can be used to connect these pipeline pieces with each other, such as push on, flanges, and welding techniques, as shown in Figure 3. In all cases, a small gap tolerance will appear between two pieces.

**Figure 4:**
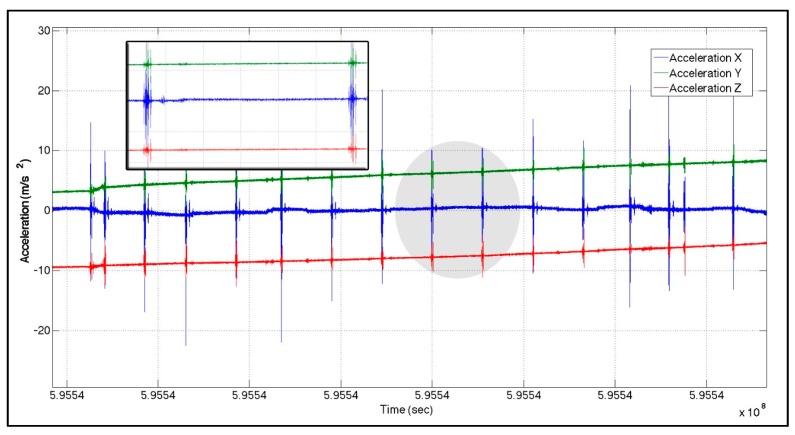
Accelerometer output—MEMS (SiIMU02).

**Figure 5:**
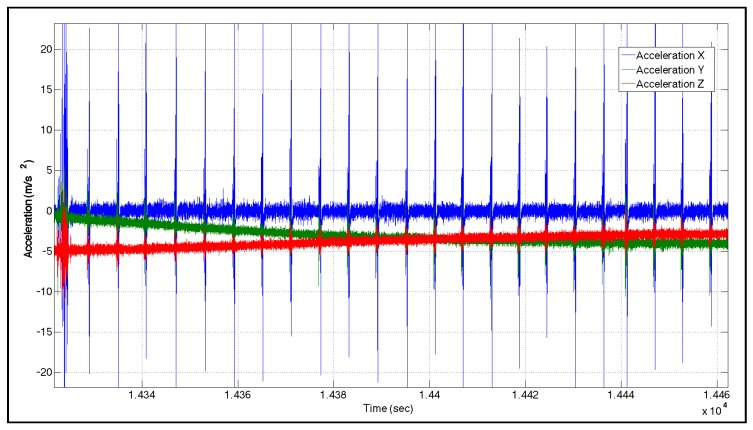
Accelerometer output—LN200.

The connection point between two pipelines is called the pipeline junction/joint. These junctions can be detected using magnetic flux leakage (MFL) and electromagnetic acoustic transducers for pipeline analysis purposes. However, due to sudden vibrations of the pig while passing through pipeline junctions, INS sensors are sensitive enough to capture these junctions. Sample accelerometer pipeline data outputs for a MEMS-based IMU (SiIMU02) and a high-end tactical grade IMU (LN200) are illustrated in Figure 4 and Figure 5, respectively. As seen, repetitive pattern spikes appear. Studying different IMUs signals collected from different pigs for different pipelines trajectories shows a repetitive signal/pattern in all the accelerometers’ signals. Taking into consideration the speed of the pig, the distance between two spikes is equal to the length of the fitting or the pipeline pieces. Therefore, the pattern represents the pipeline junction (PLJ). Such information is used as a new constraint to the estimation techniques (as explained in Section 3.2).

### 2.2. Detecting Pipeline Junctions

To detect the spikes in the signal, different methods have been used in the signal processing literature. In this paper, a discrete wavelet transformation (DWT) has been used to detect these spikes due to its simple implementation. The definition of wavelet transform can be written as:

(1) $$X_{w}\left(a,b\right)=\frac{1}{\sqrt{b}}\int^{\text{​}}x\left(t\right)M\left(\frac{t-a}{b}\right)dt$$

**Figure 6:**
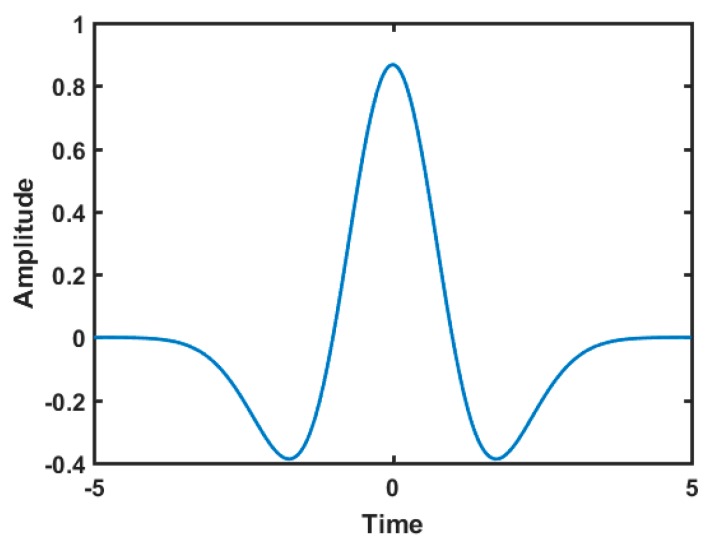
Mexican Hat mother wavelet.

Here x(.) is the input, and M(.) is the mother wavelet. The parameters (a) is real number that represent a time location. (b) is a positive real number that represents the scaling. Many types of mother wavelets can be used in signal pattern detection applications, such as the Haar basis and Mexican Hat. Figure 6 illustrates the Mexican Hat mother wavelet that has the following model:

(2) $$M\left(t\right)=\frac{2^{\frac{5}{4}}}{\sqrt{3}}\left(1-2\pi t^{2}\right)e^{-{\pi t}^{2}}$$

**Figure 7:**
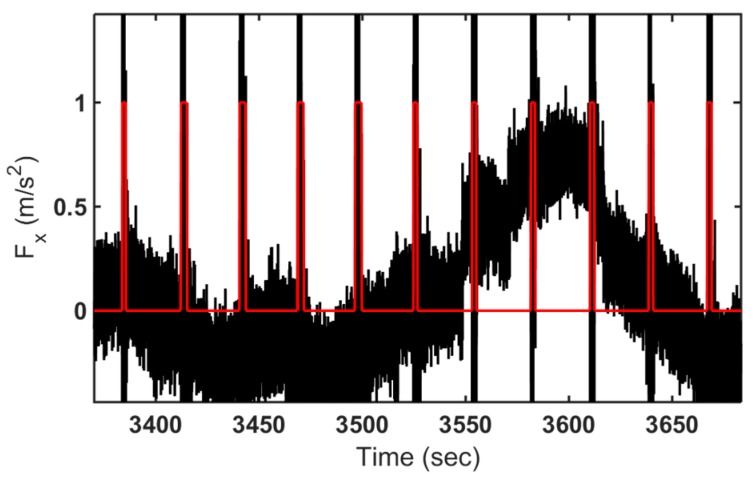
Pattern detected using WL.

The Mexican Hat mother wavelet has been applied to detect the signal patterns (pipeline junctions) as shown in Figure 7. For more details about the wavelet detection technique, please refer to [10,11,12,13,14].

Since these patterns represent the pipeline junctions, a constraint is introduced here and called Pipeline Junction Constraint (PJC). This constraint fixes both the heading and pitch angles during the movement inside the pipeline piece (not during the junctions) [15]. New measurement model for PJC will be introduced in Section 3.2. It is noticeable that roll angle is free to rotate along the pipeline axis. Therefore, roll angle will not be included in PJC measurement model.

Until now, we assumed that the period between two junctions represents a straight pipe. However, due to the fittings (*i.e.*, bends), this assumption cannot be considered true all the time. Therefore, the PJC model will be supported by a bend detection algorithm (BDA) to detect the bends and disable the PJC model during the bend periods. BDA is introduced in the next section.

Finally, it is useful to mention that any junction detection function should be executed prior to the navigation offline process to save the pipeline junctions file. This file will be used as an input to the navigation offline process.

### 2.3. Bend Detection Algorithm (BDA)

**Figure 8:**
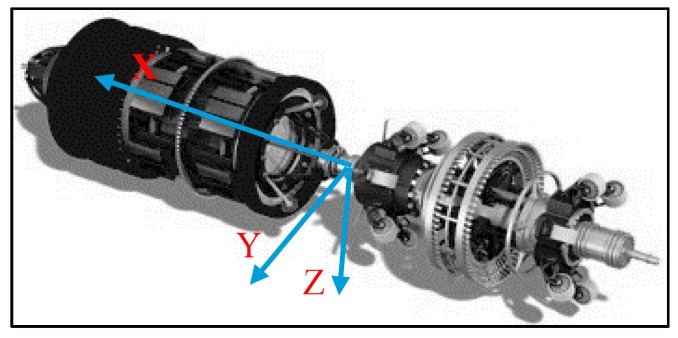
Pig defined axes.

The used IMU has three gyroscopes that measure and record the angular rates $\left(\omega_{x},\omega_{y},\omega_{z}\right)$ of pig’s motion around three axes x, y, and z, respectively. The axes are defined as shown in Figure 8. The *x*-axis angular rate $\left(\omega_{x}\right)$ measures the rate change of the roll angle, while $\omega_{y}$ and $\omega_{z}$ measure the rate change of the pitch and heading angles, respectively.

To detect whether the pig is located inside a fitting (bend), the change in heading and/or pitch angles of the pig in motion should not exceed a certain threshold $\left(C_{th}\right)$. This threshold can be selected by sensor calibration. The change rate for both the y and z axes are monitored at every epoch. The change of the angular velocity magnitude can be compared to the selected threshold $\left(C_{th}\right)$. For simplicity, both values are merged as follows:

(3) $$\omega_{R}=\sqrt{\omega_{y}^{2}+\omega_{z}^{2}}$$

**Figure 9:**
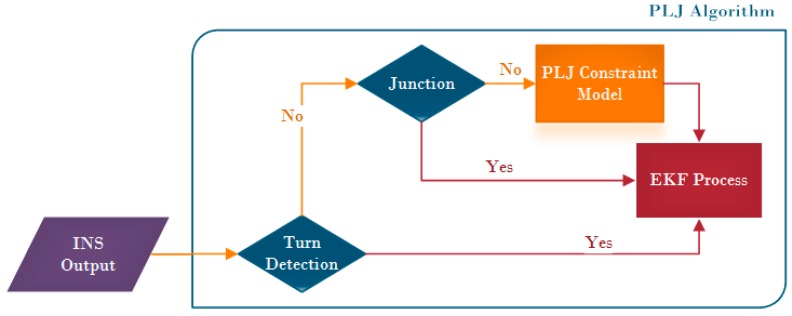
Algorithm for corrected state estimation.

Here $\omega_{R}$ is called a resultant angular rate. Selecting the threshold is the most important part in this method. By plotting different resultant angular rates for different IMUs, it has been noticed that the best value to select $\left(C_{th}\right)$ is the mean value of the static period (during the calibration time). Checking the condition of this threshold should be done at every iteration and before applying the PJC constraints as shown in Figure 9.

**Figure 10:**
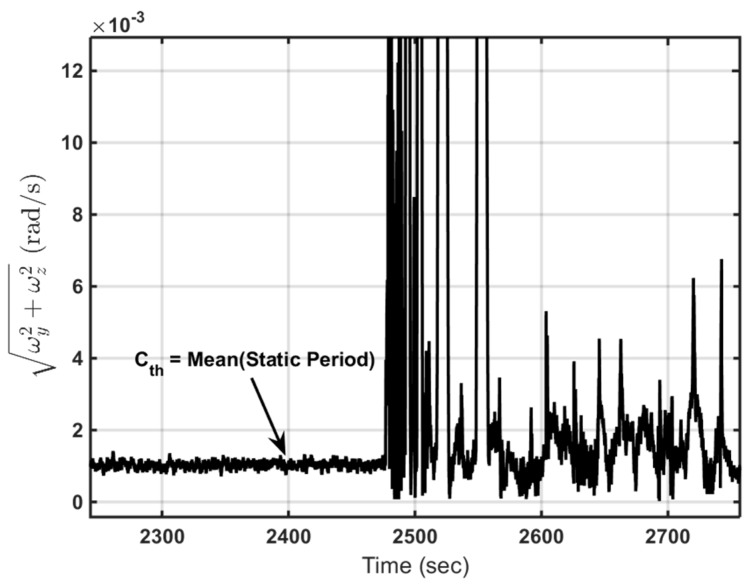
Selecting threshold criteria.

Figure 10 illustrates the $\omega_{R}$ values that have been calculated before compensating for gyro biases and scale factors. Threshold $\left(C_{th}\right)$ can be selected as the mean value of all resultant angular rates of $\omega_{y}$ and $\omega_{z}$ during the static period of the pig before it begins moving.

## 3. Methodology

To cater for all three-dimensional dynamics of the pig motion, a total of six sensors are used in a full IMU, which comprises three gyroscopes and three accelerometers. In addition to the IMU, the odometer is used to measure the displaced travel distance of the pig. AGMs and their measurement model will be shown in this section, although the target is to use the fewest number of AGMs as possible.

### 3.1. Dynamic Error Model

A MEMS-based IMU was the main sensor used to collect the pig’s motion data. EKF was used as an estimation technique to overcome the poor performance and non-linearity of the dynamic system in this work. Both dynamic and measurement models developed in this section to estimate the states of the system. The state vector to be estimated was designed to include the errors associated with the position, velocity, attitude, and the stochastic bias errors associated with the gyroscopes and accelerometers. The state vector was defined as follows [16]:

(4) $$\delta x={\left[\delta r\;\delta v\;\delta \varepsilon \;\delta b_{g}\;\delta b_{a}\right]}^{T}$$

where:

δr Position error (3×1)
δv Velocity error (3×1)
δε Attitude error (3×1)
$\delta b_{g}$ Gyroscope bias error (3×1)
$\delta b_{a}$ Accelerometer bias error (3×1).

The dynamic model is non-linear and can be represented in discrete form as follows [17,18]:

(5) $$x_{k+1}=f\left(x_{k},k\right)+g\left(x_{k},k\right)w_{k}$$

where f is the dynamic model, g is the stochastic model, and w is the process noise.

The linearized error state system model can be expressed as:

(6) $$\delta x_{k+1}=\Phi_{k}\delta x_{k}+G_{k}w_{k}$$

where:

$\delta x_{k+1}$: is the (15×1) state vector
$\Phi_{k}$: is the (15×15) transition matrix
$G_{k}$: is the (15×1) noise distribution matrix
$w_{k}$: is the unit-variance white Gaussian noise.

By applying Taylor series expansion and ignoring the higher order terms, the linearized system model in the local level frame (LLF), represented as north, east, and down (NED), can be expressed as follows:

(7) $$\delta x_{k+1}=\left[\begin{array}{ccccc} I & F_{1} & 0 & 0 & 0\\ 0 & I & F_{2} & 0 & F_{3}\\ 0 & F_{4} & I & F_{3} & 0\\ 0 & 0 & 0 & F_{5} & 0\\ 0 & 0 & 0 & 0 & F_{6} \end{array}\right]\left[\begin{array}{c} \delta r_{k}\\ \delta v_{k}\\ \delta \varepsilon \\ \delta b_{g}\\ \delta b_{a} \end{array}\right]+\left[\begin{array}{c} \sigma_{r}\\ \sigma_{v}\\ \sigma_{\varepsilon }\\ F_{7}\\ F_{8} \end{array}\right]w_{k}$$

where:

(8) $$\delta r_{k}={\left[\begin{array}{ccc} \delta \varphi_{k}, & \delta \lambda_{k}, & \delta h_{k} \end{array}\right]}^{T},\;\delta v_{k}={\left[\begin{array}{ccc} \delta v_{k}^{N}, & \delta v_{k}^{E}, & \delta v_{k}^{D} \end{array}\right]}^{T}\;\delta \varepsilon_{k}={\left[\begin{array}{ccc} \delta r_{k}, & \delta p_{k}, & \delta A_{k} \end{array}\right]}^{T},\;\delta b_{g}={\left[\begin{array}{ccc} \delta b_{gx}, & \delta b_{gy}, & \delta b_{gz} \end{array}\right]}^{T}\;\delta b_{a}={\left[\begin{array}{ccc} \delta b_{ax}, & \delta b_{ay}, & \delta b_{az} \end{array}\right]}^{T}$$

(9) $$F_{1}=\left[\begin{array}{ccc} \frac{1}{R_{M}+h} & 0 & 0\\ 0 & \frac{1}{\left(R_{N}+h\right)\cos\varphi } & 0\\ 0 & 0 & -1 \end{array}\right].\Delta t$$

(10) $$F_{2}=\left[\begin{array}{ccc} 0 & f_{u} & -f_{n}\\ -f_{u} & 0 & f_{e}\\ f_{n} & -f_{e} & 0 \end{array}\right].\Delta t$$

(11) $$F_{3}=\left[\begin{array}{ccc} R_{11} & R_{12} & R_{13}\\ R_{21} & R_{22} & R_{23}\\ R_{31} & R_{32} & R_{33} \end{array}\right].\Delta t$$

(12) $$F_{3}=\left[\begin{array}{ccc} R_{11} & R_{12} & R_{13}\\ R_{21} & R_{22} & R_{23}\\ R_{31} & R_{32} & R_{33} \end{array}\right].\Delta t$$

(13) $$F_{4}=\left[\begin{array}{ccc} \frac{1}{R_{M}+h} & 0 & 0\\ 0 & \frac{-1}{R_{N}+h} & 0\\ 0 & \frac{-\tan\varphi }{R_{N}+h} & 0 \end{array}\right].\Delta t$$

(14) $$F_{5}=\left[\begin{array}{ccc} -\beta_{\omega x} & 0 & 0\\ 0 & -\beta_{\omega y} & 0\\ 0 & 0 & -\beta_{\omega z} \end{array}\right].\Delta t$$

(15) $$F_{6}=\left[\begin{array}{ccc} -\beta_{fx} & 0 & 0\\ 0 & -\beta_{fy} & 0\\ 0 & 0 & -\beta_{fz} \end{array}\right].\Delta t$$

(16) $$\sigma_{r}=\left[\begin{array}{c} \sigma_{\varphi }\\ \sigma_{\lambda }\\ \sigma_{h} \end{array}\right],\;\sigma_{v}=\left[\begin{array}{c} \sigma_{v^{n}}\\ \sigma_{v^{e}}\\ \sigma_{v^{d}} \end{array}\right],\;\sigma_{\varepsilon }=\left[\begin{array}{c} \sigma_{r}\\ \sigma_{p}\\ \sigma_{A} \end{array}\right]$$

(17) $$F_{7}=\left[\begin{array}{c} \sqrt{2\beta_{\omega x}\sigma_{\omega x}^{2}}\\ \sqrt{2\beta_{\omega y}\sigma_{\omega y}^{2}}\\ \sqrt{2\beta_{\omega z}\sigma_{\omega z}^{2}} \end{array}\right],\;F_{8}=\left[\begin{array}{c} \sqrt{2\beta_{fx}\sigma_{fx}^{2}}\\ \sqrt{2\beta_{fy}\sigma_{fy}^{2}}\\ \sqrt{2\beta_{fz}\sigma_{fz}^{2}} \end{array}\right]$$

where:

β Reciprocal of the correlation time of the process
$\sigma^{2}$ Variance of the white noise associated with the random process
$R_{M}$ Meridian radius of curvature (North-South)
$R_{N}$ Prime vertical radius of curvature of the Earth’s surface (East-West)
φ, λ, h Latitude, longitude and height, respectively
$f^{n},f^{e},f^{d}$ Specific forces in east, north and up directions, respectively.
$R_{ij}$ Rotation matrix $\left(R_{b}^{l}\right)$ elements from body to local level frame.
Δt Rate change of time.

The linearized measurement error model can be expressed as:

(18) $$\delta z_{k}=H\delta x_{k}+\delta \nu_{k}$$

Here H is the design matrix and ν is the measurement noise. Both process and measurement noises are assumed to be white and uncorrelated to each other. Readers can refer to [19] for more details about measurement models. In this work, a new measurement model has been developed specifically for pipeline navigation. The mathematical equations will be demonstrated in the next section.

### 3.2. Pitch & Heading Measurement Model

The rotation matrix (direct cosine matrix—DCM) is updated at every epoch. The attitude angles (roll, pitch, and heading) can be calculated using this matrix. Heading and pitch angles of the pig in the pipeline are computed from the elements of the following (DCM) [19]:

(19) $${\hat{R}}_{v}^{l}={\hat{R}}_{b}^{l}{\left(R_{b}^{v}\right)}^{T}=\left[I-\Psi \right]R_{b}^{l}{\left(R_{b}^{v}\right)}^{T}=\left[\begin{array}{ccc} {\hat{a}}_{11} & {\hat{a}}_{12} & {\hat{a}}_{13}\\ {\hat{a}}_{21} & {\hat{a}}_{22} & {\hat{a}}_{23}\\ {\hat{a}}_{31} & {\hat{a}}_{32} & {\hat{a}}_{33} \end{array}\right]$$

where $\left(R_{j}^{k}\right)$ represents the DCM from (j) to (k) frames, (b,l,v) represent the IMU body, local level (navigation), and vehicle (pig) frames, respectively. The rotation matrix from the body to vehicle (pig) frame is calculated after installing the IMU in the pig. $R_{b}^{v}$ is constant and does not change. (Ψ) represents the skew-symmetric matrix of the rotation vector pertaining to the error of the attitude DCM and can be expressed as follows:

(20) $$\Psi =\left[\begin{array}{ccc} 0 & -\delta A & \delta p\\ \delta A & 0 & -\delta r\\ -\delta p & \delta r & 0 \end{array}\right]$$

Let $b_{ij}$ and $c_{ij}$ represent the $ij^{th}$ elements of $R_{b}^{v}$ and $R_{b}^{l}$, respectively. The $R_{b}^{l}$ can be written as follows:

(21) $$R_{b}^{l}=\left[\begin{array}{ccc} \cos p\cos A & -\cos r\sin A+\sin r\sin p\cos A & \sin r\sin A+\cos r\sin p\cos A\\ \cos p\sin A & \cos r\cos A+\sin r\sin p\sin A & -\sin r\cos A+\cos r\sin p\sin A\\ -\sin p & \sin r\cos p & \cos r\cos p \end{array}\right]$$

From Equation (19), the required ${\hat{a}}_{ij}$ elements to be used for heading and pitch angles calculation can be expressed as follows:

(22) $${\hat{a}}_{11}=b_{11}\left(c_{11}+c_{21}\delta A-c_{31}\delta p\right)+b_{12}\left(c_{12}+c_{22}\delta A-c_{32}\delta p\right)+b_{13}\left(c_{13}+c_{23}\delta A-c_{33}\delta p\right)\;{\hat{a}}_{21}=b_{11}\left(c_{21}+c_{31}\delta r-c_{11}\delta A\right)+b_{12}\left(c_{22}+c_{32}\delta r-c_{12}\delta A\right)+b_{13}\left(c_{23}+c_{33}\delta r-c_{13}\delta A\right)\;{\hat{a}}_{31}=b_{11}\left(c_{31}+c_{11}\delta p-c_{21}\delta r\right)+b_{12}\left(c_{32}+c_{12}\delta p-c_{22}\delta r\right)+b_{13}\left(c_{33}+c_{13}\delta p-c_{23}\delta r\right)\;{\hat{a}}_{32}=b_{21}\left(c_{31}+c_{11}\delta p-c_{21}\delta r\right)+b_{22}\left(c_{32}+c_{12}\delta p-c_{22}\delta r\right)+b_{23}\left(c_{33}+c_{13}\delta p-c_{23}\delta r\right)\;{\hat{a}}_{33}=b_{31}\left(c_{31}+c_{11}\delta p-c_{21}\delta r\right)+b_{32}\left(c_{32}+c_{12}\delta p-c_{22}\delta r\right)+b_{33}\left(c_{33}+c_{13}\delta p-c_{23}\delta r\right)$$

From Equation (21), heading and pitch can be calculated as follows:

(23) $$A=\tan^{-1}\frac{\sin A}{\cos A}=\tan^{-1}\frac{a_{21}}{a_{11}}$$

(24) $$p=\tan^{-1}\frac{sinp}{cosp}=\tan^{-1}\frac{-a_{31}}{\sqrt{a_{32}^{2}+a_{33}^{2}}}$$

Similarly, the estimated heading and pitch angles can be calculated from Equation (19) as follows:

(25) $$\hat{A}=\tan^{-1}\frac{\sin\hat{A}}{\cos\hat{A}}=\tan^{-1}\frac{{\hat{a}}_{21}}{{\hat{a}}_{11}}$$

(26) $$\hat{p}=\tan^{-1}\frac{sin\hat{p}}{cos\hat{p}}=\tan^{-1}\frac{-{\hat{a}}_{31}}{\sqrt{{\hat{a}}_{32}^{2}+{\hat{a}}_{33}^{2}}}$$

Ideally as per the algorithm assumption, the heading and pitch angles do not change in the pipeline piece; the change in these angles should be zero. Therefore, the approximated changes of heading and pitch angles model can be expressed as follows:

(27) $$\delta z_{p,A}^{v}=\hat{\epsilon }-\tilde{\epsilon }$$

where $\hat{\epsilon }$ is the computed heading and pitch vector, and $\tilde{\epsilon }$ is the measured heading and pitch vector.

The measurement design matrix can be expressed as follows:

(28) $$H_{p,A}=\left[\begin{array}{ccc} \frac{\partial \hat{p}}{\partial \delta r} & \frac{\partial \hat{p}}{\partial \delta p} & \frac{\partial \hat{p}}{\partial \delta A}\\ \frac{\partial \hat{A}}{\partial \delta r} & \frac{\partial \hat{A}}{\partial \delta p} & \frac{\partial \hat{A}}{\partial \delta A} \end{array}\right]$$

The design matrix elements are expanded in the Appendix. Finally, the innovation sequence of EKF at each epoch can be calculated as follows:

(29) $$e_{k}=\delta z_{p,A}^{v}-\;H_{p,A}\delta x$$

where $\delta x_{k}$ represents the error state vector ${\left[\begin{array}{ccc} \delta r & \delta p & \delta A \end{array}\right]}^{T}$.

## 4. Results

This section introduces the equipment used and describes the pipeline inspection test performed to assess the efficacy of the PLJ algorithm. The results of the proposed method—Pipeline junctions integration—Will be discussed in detail and compared with the results of the traditional method of the EKF-based odometer/AGM integration for pipeline navigation. In figures and tables, EKF/PLJ will be referred to as the new developed method, while EKF will be referred to as the normal EKF method. Please note that odometer and AGM (if available) are used in both methods. The developed method was examined through real pipeline inspection trajectories, using as few AGMs as possible.

The pig’s data and the reference trajectory have been provided by M/s ROSEN. A SiIMU02 (by UTC Aerospace Systems, Brea, CA, USA) MEMS-based inertial sensor was used for the experiment. SiIMU02 is a six degree of freedom inertial system that uses solid-state devices to measure the angular rate and linear acceleration. Table 1 shows the IMU specifications. The forward velocity time stamp was synchronized with the IMU data. The odometer standard deviation (STD) is 0.15 m/s.

One point worth noting is that the pig operator did not provide the AGMs’ positions, however, they provided the full reference trajectory. As a result, an artificial AGMs were extracted from the true reference trajectory. The total pig journey distance is almost 3 km over a total travel time of 1 h.

For comparison purposes, the proposed algorithm was applied for two different scenarios. In both scenarios, the position of the first and last point of the trajectory is known. The first point represents the pig pipeline inlet and the last point is the pig pipeline outlet.

• Scenario #1: Processed IMU & odometer data using one AGM (after 20 min)
• Scenario #2: Processed IMU & odometer data using no AGM.

### 4.1. Scenario #1

**Figure 11:**
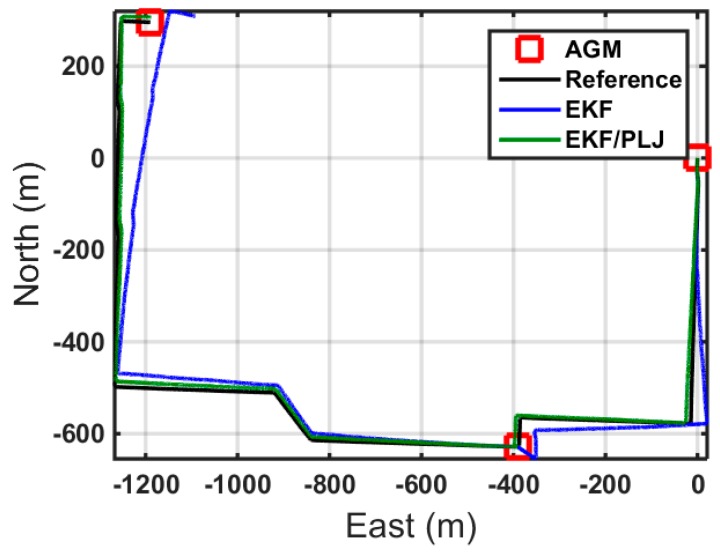
Trajectories—Case #1.

**Figure 12:**
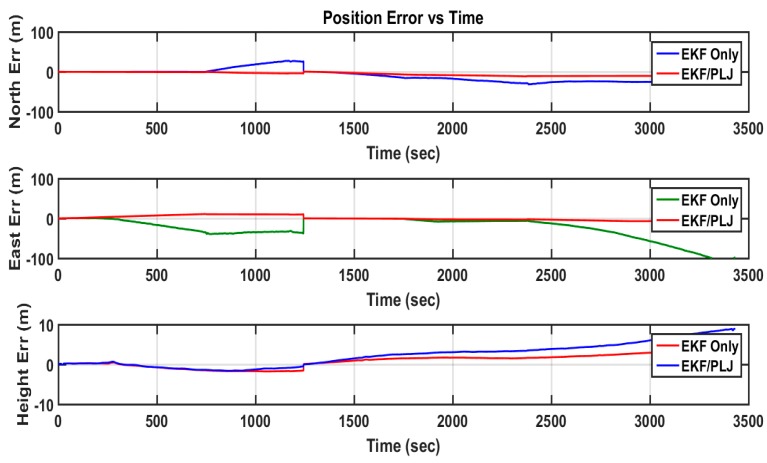
Position Errors-EKF & EKF/PLJ Scenario #1.

**Figure 13:**
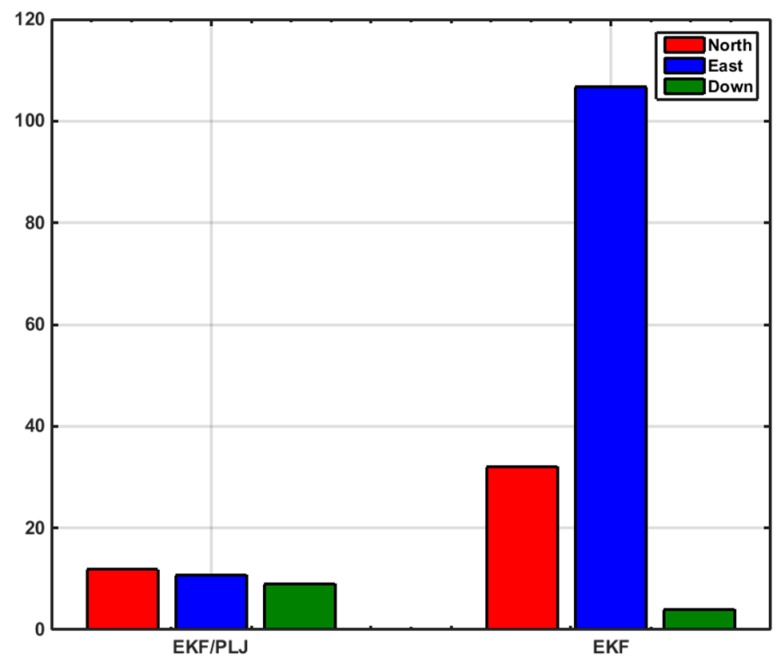
Maximum Position Error—Scenario #1.

**Figure 14:**
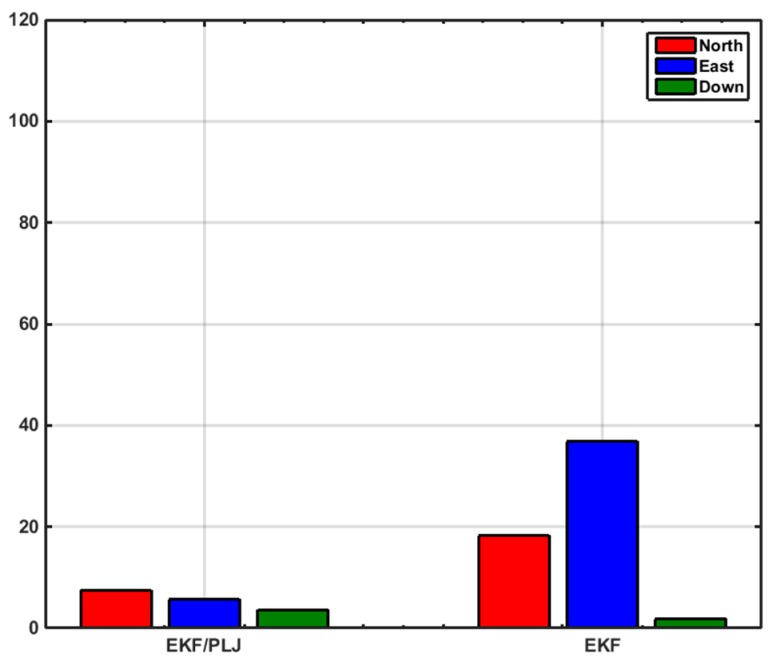
RMS Position Errors—Scenario #1.

In this scenario, one AGM was added after 20 min of motion to provide position update (CUPT) to the navigation algorithm. Figure 11 shows the solutions of the EKF-based and EKF/PLJ-based, where both solutions are compared against the reference trajectory. The EKF/PLJ proposed solution showed an improvement over the standalone EKF solution, which is clearly noticed in Figure 12. The figures illustrate the north, east, and height position errors for both methods. The errors in the north and east direction are clearly smaller in the EKF/PLJ algorithm. However, the height error is slightly smaller using EKF only. The maximum and RMS position errors (in meters) for each solution are shown by bar graphs in Figure 13 and Figure 14, respectively.

### 4.2. Scenario #2

**Figure 15:**
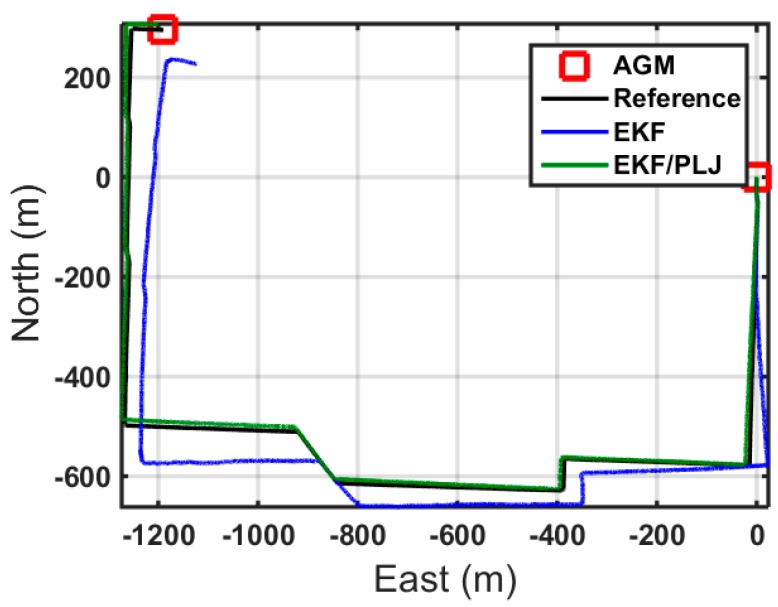
Trajectories—Scenario #2.

**Figure 16:**
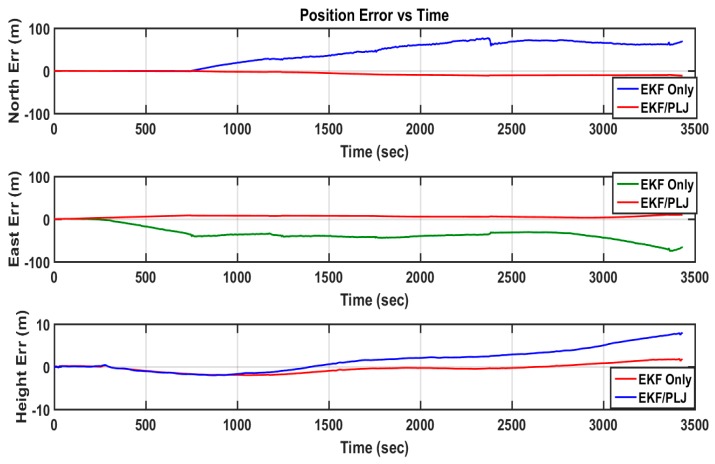
Positions Errors—EKF & EKF/PLJ Scenario #2.

Scenario #2 is similar to scenario #1, without adding any AGM. Directly from the output trajectory, the difference between both solutions is clear, as shown in Figure 15. The position errors for each method are shown in Figure 16.

**Figure 17:**
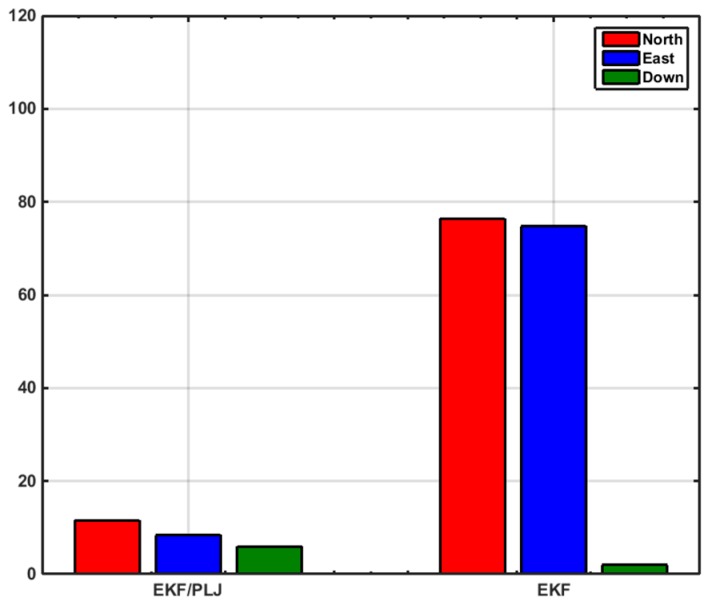
Maximum Position Errors—Scenario #2.

**Figure 18:**
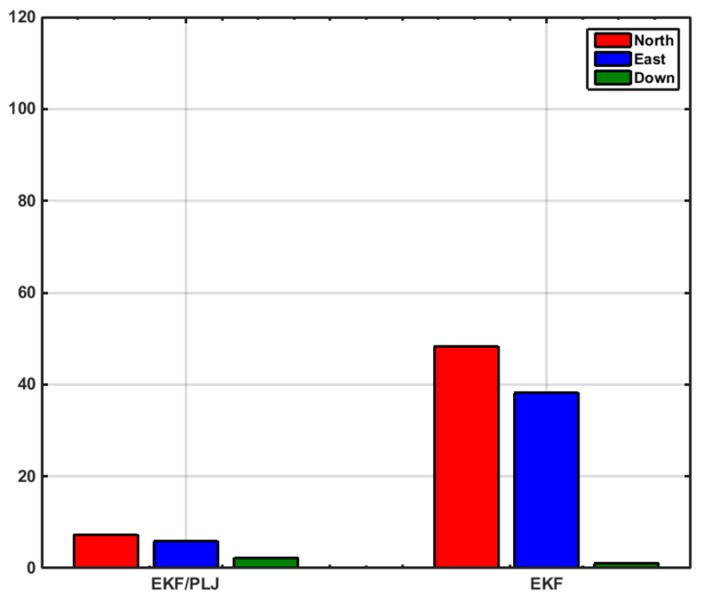
RMS Positions Errors—Scenario #2.

The bar graphs in Figure 17 and Figure 18 show that the proposed EKF/PLJ method greatly improve the accuracy of the results.

EKF/Odometer integration for pipeline navigation, using the developed method of EKF/PLJ, had a maximum position error of 11.59 m in the north direction, and EKF/odometer solution only had a maximum position error of 76.41 m in the same direction. This is an overall average improvement of 85%. Despite all the improvements in the horizontal plane (north and east directions), the above bar graphs reveal that height errors have slight increase using the new EKF/PLJ method. This increase does not affect the total solution for the pipeline trajectory, especially, if we consider that the pipeline is usually located 1–5 m below the Earth surface. Finally, the results can be summarized as shown in Table 2 and Table 3 where north, east, height maximum and RMS errors for both scenarios are shown.

## 5. Conclusions

The main objective of this work was to increase the accuracy of pipeline navigation. Based on the reality of constructing the pipelines (*i.e.*, straight shapes), and how they connect to each other, pipeline junctions have been modeled and included as new measurements to update the navigation estimation algorithm. The new developed algorithm will also lead to a reduction in the total required number of AGMs. Two different testing scenarios were discussed based on real pipeline data that was collected using a MEMS-IMU-based system. These results show that the newly proposed method is capable of reducing the trajectory navigation RMS error by around 80% over one hour of operation and without using any AGM along the pig journey.

## Figures and Tables

**Table 1:** MEMS-IMU Specifications.

	Gyroscope	Accelerometer
Bias Repeatability (1σ)	≤100°/h	≤ 10 mg
Bias Instability	≤6°/h	≤ 0.5 mg
Random Walk	$\leq 0.5{}^{\circ}/\sqrt{h}$	$\leq 0.5\;m/s/\sqrt{h}$
Size (mm)	Diameter (55.88)	
	Depth (35.56)	

**Table 2:** North, East & Height Errors.

Maximum Error				
Method		North (m)	East (m)	Height (m)
Scenario #1	EKF	31.99	72.89	3.28
	EKF/PLJ	11.83	10.77	7.03
Scenario #2	EKF	76.41	51.94	1.99
	EKF/PLJ	11.59	8.48	5.99

**Table 3:** North, East & Height Errors.

RMS Error				
Method		North (m)	East (m)	Height (m)
Scenario #1	EKF	17.7	25.05	1.61
	EKF/PLJ	7.11	5.80	2.91
Scenario #2	EKF	46.72	34.58	1.03
	EKF/PLJ	7.35	5.91	2.31

## Appendix A

The elements in Equation (28) can be calculated as follows:

$$H_{p,A}=\left[\begin{array}{ccc} \frac{\partial \hat{p}}{\partial \delta r} & \frac{\partial \hat{p}}{\partial \delta p} & \frac{\partial \hat{p}}{\partial \delta A}\\ \frac{\partial \hat{A}}{\partial \delta r} & \frac{\partial \hat{A}}{\partial \delta p} & \frac{\partial \hat{A}}{\partial \delta A} \end{array}\right]$$

$$\frac{\partial \hat{p}}{\partial \delta r}=\frac{\left(\frac{EQ_{5}}{\sqrt{EQ_{7}}}-\frac{\left[2\left(b_{21}c_{21}+b_{22}c_{22}+b_{23}c_{23}\right)EQ_{9}+2\left(b_{31}c_{21}+b_{32}c_{22}+b_{33}c_{23}\right)EQ_{8}\right]EQ_{4}}{EQ_{3}}\right)EQ_{7}}{EQ_{1}}$$

$$\frac{\partial \hat{p}}{\partial \delta p}=\frac{\left(\frac{EQ_{6}}{\sqrt{EQ_{7}}}-\frac{\left[2\left(b_{21}c_{11}+b_{22}c_{12}+b_{23}c_{13}\right)EQ_{9}+2\left(b_{31}c_{11}+b_{32}c_{12}+b_{33}c_{13}\right)EQ_{8}\right]EQ_{4}}{EQ_{3}}\right)EQ_{7}}{EQ_{1}}$$

$$\frac{\partial \hat{p}}{\partial \delta A}=0$$

$$\frac{\partial \hat{A}}{\partial \delta r}=\frac{\left(b_{11}c_{11}+b_{12}c_{12}+b_{13}c_{13}\right)\left(b_{11}c_{31}+b_{12}c_{32}+b_{13}c_{33}\right)}{{\left(b_{11}c_{11}+b_{12}c_{12}+b_{13}c_{13}\right)}^{2}+{\left(b_{11}c_{21}+b_{12}c_{22}+b_{13}c_{33}\right)}^{2}}$$

$$\frac{\partial \hat{A}}{\partial \delta p}=\frac{\left(b_{11}c_{21}+b_{12}c_{22}+b_{13}c_{23}\right)\left(b_{11}c_{31}+b_{12}c_{32}+b_{13}c_{33}\right)}{{\left(b_{11}c_{11}+b_{12}c_{12}+b_{13}c_{13}\right)}^{2}+{\left(b_{11}c_{21}+b_{12}c_{22}+b_{13}c_{33}\right)}^{2}}$$

$$\frac{\partial \hat{A}}{\partial \delta A}=\frac{\left(\frac{{\left(b_{11}c_{21}+b_{12}c_{22}+b_{13}c_{23}\right)}^{2}}{{\left(b_{11}c_{11}+b_{12}c_{12}+b_{13}c_{13}\right)}^{2}}+1\right){\left(b_{11}c_{11}+b_{12}c_{12}+b_{13}c_{13}\right)}^{2}}{{\left(b_{11}c_{11}+b_{12}c_{12}+b_{13}c_{13}\right)}^{2}+{\left(b_{11}c_{21}+b_{12}c_{22}+b_{13}c_{33}\right)}^{2}}=-1$$

where

$$EQ_{1}={\left(b_{11}c_{31}+b_{12}c_{32}+b_{13}c_{33}\right)}^{2}+{\left(b_{21}c_{31}+b_{22}c_{32}+b_{23}c_{33}\right)}^{2}+{\left(b_{31}c_{31}+b_{32}c_{32}+b_{33}c_{33}\right)}^{2}$$

$$EQ_{2}={\left(b_{11}c_{11}+b_{12}c_{12}+b_{13}c_{13}\right)}^{2}+{\left(b_{11}c_{21}+b_{12}c_{22}+b_{13}c_{23}\right)}^{2}$$

$$Q_{3}=2\sqrt{{\left({\left(b_{21}c_{31}+b_{22}c_{32}+b_{23}c_{33}\right)}^{2}+{\left(b_{31}c_{31}+b_{32}c_{32}+b_{33}c_{33}\right)}^{2}\right)}^{3}}$$

$$EQ_{4}=b_{11}c_{31}+b_{12}c_{32}+b_{13}c_{33}$$

$$EQ_{5}=b_{11}c_{21}+b_{12}c_{22}+b_{13}c_{23}$$

$$Q_{6}=b_{11}c_{11}+b_{12}c_{12}+b_{13}c_{13}$$

$$EQ_{7}=EQ_{8}^{2}+EQ_{9}^{2}$$

$$EQ_{8}=b_{31}c_{31}+b_{32}c_{32}+b_{33}c_{33}$$

$$EQ_{9}=b_{21}c_{31}+b_{22}c_{32}+b_{23}c_{33}$$
